# A UoI-Optimal Policy for Timely Status Updates with Resource Constraint

**DOI:** 10.3390/e23081084

**Published:** 2021-08-20

**Authors:** Lehan Wang, Jingzhou Sun, Yuxuan Sun, Sheng Zhou, Zhisheng Niu

**Affiliations:** Beijing National Research Center for Information Science and Technology, Department of Electronic Engineering, Tsinghua University, Beijing 100084, China; wang-lh19@mails.tsinghua.edu.cn (L.W.); sunjz18@mails.tsinghua.edu.cn (J.S.); sunyuxuan@tsinghua.edu.cn (Y.S.); niuzhs@tsinghua.edu.cn (Z.N.)

**Keywords:** age of information, constrained Markov decision process, reinforcement learning, context-awareness, timely status updates

## Abstract

Timely status updates are critical in remote control systems such as autonomous driving and the industrial Internet of Things, where timeliness requirements are usually context dependent. Accordingly, the Urgency of Information (UoI) has been proposed beyond the well-known Age of Information (AoI) by further including context-aware weights which indicate whether the monitored process is in an emergency. However, the optimal updating and scheduling strategies in terms of UoI remain open. In this paper, we propose a UoI-optimal updating policy for timely status information with resource constraint. We first formulate the problem in a constrained Markov decision process and prove that the UoI-optimal policy has a threshold structure. When the context-aware weights are known, we propose a numerical method based on linear programming. When the weights are unknown, we further design a reinforcement learning (RL)-based scheduling policy. The simulation reveals that the threshold of the UoI-optimal policy increases as the resource constraint tightens. In addition, the UoI-optimal policy outperforms the AoI-optimal policy in terms of average squared estimation error, and the proposed RL-based updating policy achieves a near-optimal performance without the advanced knowledge of the system model.

## 1. Introduction

With the development of 5G and the Internet of Things (IoT), requirements for wireless communication have shifted from merely providing communication channels to covering the entire process of various IoT applications, e.g., autonomous vehicle [1] and virtual reality (VR) [2], where sensing, communication, computation, and control form a closed loop. Therefore, in addition to the communication delay, it is necessary to consider the information delay counted from the generation of the state information to the execution, namely the timeliness of information. For this purpose, Age of Information (AoI) has been proposed, which is defined as the time elapsed since the generation time of the latest received packets [3]. Due to its concise definition and clear physical meaning, AoI has been widely used for the design of scheduling and updating policies in remote estimation [4,5,6] and wireless communication networks [7,8,9,10,11,12]. Most existing works focus on optimizing average AoI or peak age. In [13], the authors claim that minimizing average age cannot satisfy the requirements for ultra-reliable low-latency communication (URLLC) and study the tail distribution of AoI. The violation probability for peak age is derived in [14] and the stationary distribution of AoI is studied in [15].

Nevertheless, the AoI still has some limitations. First, it fails to measure the nonlinear performance degradation caused by information staleness. In [16,17,18,19], nonlinear age penalty functions were introduced to solve this problem. Meanwhile, the Age of Synchronization (AoS) [20] and Age of Incorrect Information (AoII) [21] are defined to associate information freshness with the content of information. AoS is the time elapsed since the information at the receiver becomes desynchronized with the actual status of the monitored process. AoII is defined as the product of an increasing time penalty function and a penalty function of the estimation error. In addition, the status of heterogeneous data sources may change at different rates. A fast-changing process may require information with a lower age. However, age is independent of the changing rate and thus is not proper in the cases when heterogeneous data sources are jointly considered. To solve this problem, weighted age was introduced in [22,23] to distinguish important monitored processes. In [24], the metric based on information theory is proposed as a replacement of the time-based metric, AoI, to characterize the changing rate. In [5], the authors claim that minimizing age is not equivalent to minimizing the estimation error in a remote estimation problem and propose an effective age to solve this problem [25].

Practical systems (e.g., V2X-communication systems) may have different requirements for information freshness with different contexts. The context refers to all environmental factors that affect the requirement for information freshness. Therefore, resources should be reserved for frequent status updates in emergency to ensure safety.

However, the timeliness metrics mentioned above pay no attention to the significance of context information. To solve this problem, Urgency of Information (UoI) has been proposed in [26,27,28] to measure the influence of inaccurate information on performance under different contexts. To be specific, UoI uses a time-variant context-aware weight ω(t) to distinguish different contexts. A higher ω(t) indicates that the system is in more urgent situations (e.g., when a vehicle is approaching an intersection or overtaking) and therefore requires frequent updates. For example, when a vehicle passes through an intersection, the context-aware weight increases as the distance between the vehicle and the center of the intersection decreases. Meanwhile, the estimation error Q(t) is introduced to measure the information inaccuracy, which is defined as the difference between the actual status and the estimated status at the receiver. The larger the absolute value of Q(t) is, the less accurate the estimated status is. Therefore, UoI is defined as the product of context-aware weight and a cost function of the estimation error Q(t):

(1) F(t)=ω(t)δ(Q(t)).

In discrete-time systems, the estimation error Q(t) is:

(2) $$Q\left(t\right)=\sum_{\tau =g(t)}^{t-1}A\left(\tau \right),$$

where g(t) is the generation time of the latest status update at the receiver and A(t) is the increment in estimation error in time slot *t*. Specifically, if the context-aware weight is time-invariant (i.e., ω(t)=1), and A(t)=1 as well as δ(Q(t))=Q(t), UoI is the same as AoI. If the context-aware weight is process-dependent, UoI can represent weighted age. If the cost function δ(Q(t)) is nonlinear, UoI can represent the nonlinear age penalty function. For example, when the outdated information is worthless, e.g., the information is about sales that expire after some time [29], then the shifted unit step cost function δ(Q(t))=u(Q(t)−τ), τ>0 is recommended. For the unit step function, u(x)=1 when x≥0 and otherwise u(x)=0.

In this work, we considered a single-user remote monitoring system, and the objective was to find an updating policy minimizing the average UoI over time under the constraint on average update frequency. To solve this problem, Refs. [27,30] proposed update-index-based adaptive schemes with Lyapunov optimization but did not conduct a theoretical analysis of their optimality. In addition, the constrained Markov decision process (CMDP) formulation was only used in the simulation for a numerically solved benchmark. Based on the existing works, in this paper, we theoretically analyzed the structure of the UoI-optimal policy and focused on how to derive an updating policy in an unknown environment.

The main contributions of this paper are summarized as follows.

- In contrast to [27,30], we assumed that the context-aware weight is a first-order irreducible positive recurrent Markov process or independent and identically distributed (i.i.d.) over time. We formulated the updating problem as a CMDP problem and proved the single threshold structure of the UoI-optimal policy. We then derived the policy through LP with the threshold structure and discussed the conditions that the monitored process needs to satisfy for the threshold structure.
- When the distributions of the context-aware weight and the increment in estimation error were unknown, we used model-based RL method to learn the state transitions of the whole system and derive a near-optimal RL-based updating policy.
- Simulations were conducted to verify the theoretical analysis of the threshold structure and show the near-optimal performance of the RL-based updating policy. The results indicate that: (i) the update thresholds decrease when the maximum average update frequency becomes large; (ii) the update threshold for emergency can actually be larger than that for ordinary states when the probability of transferring from emergency to ordinary states tends to 1.

The rest of this paper is organized as follows. The system model and the problem formulation are described in Section 2. In Section 3, we obtain the CMDP formulation of the problem with the given distribution of context-aware weight and prove the threshold structure of the UoI-optimal policy. The proposed model-based RL updating policy is obtained in Section 4. In Section 5, the simulation results are shown and discussed while the conclusions are drawn in Section 6.

## 2. System Model and Problem Formulation

**Figure 1:**
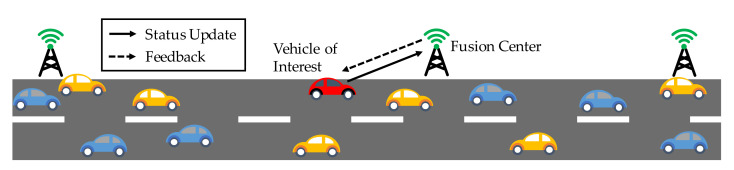
Remote control and monitoring model. The vehicle of interest is shown in red.

In this paper, we considered a remote monitoring system, in which a fusion center collects the status information (e.g., current location, velocity, information of surrounding) from a vehicle of interest via a wireless channel with limited resources, as shown in Figure 1. The whole system is considered as a discrete-time system and the status can be generated at will. Due to the limitations on the wireless resources and energy supply, there is a constraint on the average update frequency of the vehicle. The update decision in time slot *t* is denoted by U(t)∈{0,1}, where U(t)=1 means that the vehicle decides to transmit the current status to the center, and U(t)=0 denotes that the vehicle decides to stay idle.

The wireless channel is assumed as a block fading channel with successful transmission probability $p_{s}$. Let S(t)∈{0,1} be the state of the channel. S(t)=0 represents that the channel is in deep fading, and no packet can be successfully transmitted. S(t)=1 means the packets can be successfully transmitted to the center through the channel. If the center receives an update, then U(t)S(t)=1 and an ACK will be sent to the vehicle.

Let x(t) and $\hat{x}\left(t\right)$ denote the current status of the monitored vehicle and the estimated status of the vehicle at the center, and $Q\left(t\right)=x\left(t\right)-\hat{x}\left(t\right)$ denotes the estimation error. Similar to [26], we further assume that the time period of a packet transmission is less than a time slot and the estimation at the center equals the latest status information received by the center. This estimation scheme is easy to implement, theoretically tractable and has been proven to be an optimal policy that can minimize the average squared error of status estimation in a remote estimation system under energy constraints when the monitored process is a Wiener process [31]. Then, the recurrence relation of the estimation error Q(t) is:

(3) Q(t+1)=(1−U(t)S(t))Q(t)+A(t).

Equation (3) indicates that the estimation error will be the amount of variation of the monitored process from the generation time of the latest received status to the current time. The increment A(t) represents the variation of the monitored process. For example, when A(t) follows a Gaussian distribution with a mean of zero and variance of $\sigma^{2}$, represented by $N(0,\sigma^{2})$, the monitored status follows a Wiener process. When A(t) takes values from {0,1,−1} with a probability of $\left\{1-2p_{rw},p_{rw},p_{rw}\right\}$, where $0<p_{rw}<\frac{1}{2}$, then the status of the monitored source will be a one-dimensional random walk. In this paper, we assumed that the monitored status of the vehicle is a Wiener process and A(t) is i.i.d. over time. However, the increment in estimation error during a single slot cannot be infinite in practical systems. Therefore, in contrast to [27,30], we assumed that increment A(t) obeys a truncated Gaussian distribution, i.e., the probability density function (PDF) of A(t) is:

(4) $$\begin{array}{c} f_{A(t)}\left(a\right)=\frac{\frac{1}{\sigma }\phi \left(\frac{a-\mu }{\sigma }\right)}{\Phi \left(\frac{A_{max}-\mu }{\sigma }\right)-\Phi \left(\frac{-A_{min}-\mu }{\sigma }\right)}, \end{array}$$

where μ and σ are the expectation and standard deviation of increment A(t). ϕ and Φ are the PDF and the cumulative distribution function (CDF) of standard normal distribution. We also assumed $A\left(t\right)\in \left[-A_{min},A_{max}\right],A_{max}=A_{min}>0$ and μ=0.

Meanwhile, the scheduling policy of information updates should also be related to the situation and environment of the system. For example, when the system is in an emergency, it should be very sensitive to the accuracy and the delay of the status information, thus the status should be updated more frequently. Therefore, our objective is to find a policy telling the vehicle whether to transmit status information or not in each slot for a minimum average UoI over time under the constraint:

(5) $$\begin{array}{cc} \min_{U(t)} & \underset{T\to \infty }{lim sup}\frac{1}{T}E\left[\sum_{t=0}^{T-1}w\left(t\right)Q{\left(t\right)}^{2}\right]\\ s.t. & \underset{T\to \infty }{lim sup}\frac{1}{T}\sum_{t=0}^{T-1}E\left[U(t)\right]\leq \rho , \end{array}$$

where ω(t)>0 is the context-aware weight, which is independent with Q(t). ρ∈(0,1] is the maximum average update frequency. The cost function of the estimation error used here is $\delta \left(Q\left(t\right)\right)={\left(Q\left(t\right)\right)}^{2}$, which is inspired by the squared error of status estimation.

## 3. Scheduling with CMDP-Based Approach

In this section, we start by formulating problem (5) into a constrained Markov decision process (CMDP) with assumptions on the distribution of the context-aware weight. We will prove the threshold structure of the UoI-optimal updating policy and derive the optimal policy through a linear programming (LP) formulation.

### 3.1. Constrained Markov Decision Process Formulation

In the remote monitoring system, the context may be related to the distance between adjacent vehicles/mobile devices, the unexpected maneuver of the neighboring vehicles, etc. In [32], the authors prove that whether the distance between two mobile wireless devices with Ornstein–Uhlenbeck mobility is less than a certain threshold follows a first-order Markov process. When the two devices are closer, they are more interested in each other’s status information, communication and computing resources to facilitate cooperation, share resources, and avoid collisions. At this time, the transmission of status information is more urgent than when the two devices are far apart. As for the unexpected maneuver of the neighboring vehicles, it is very challenging to find a proper formulation. Instead, we assumed that such emergencies occur independently in each slot according to a certain probability. Therefore, in contrast to [27,30], we assumed that the context-aware weight ω(t) is i.i.d. over time or a first-order irreducible positive recurrent Markov process and formulated the problem (5) as a CMDP problem. The irreducible positive recurrent Markov formulation guarantees the existence of the UoI-optimal policy (see Appendix A). In this section, we will first focus on the situation where ω(t) is a first-order Markov process:

- State space: The state of the vehicle in slot *t*, denoted by s(t)=(Q(t),ω(t)), includes the current estimation error and the context-aware weight. Then, we discretize Q(t) with the step size $\Delta_{Q}>0$, i.e., the estimation error $Q\left(t\right)\in Q=\left\{0,\pm \Delta_{Q},\pm 2\Delta_{Q},\cdots ,\pm n\Delta_{Q},\cdots \right\}$. For example, when $Q\left(t\right)\in \left[n\Delta_{Q}-\frac{1}{2}\Delta_{Q},n\Delta_{Q}+\frac{1}{2}\Delta_{Q}\right)$, its value will be taken as $n\Delta_{Q}$. The smaller the step size $\Delta_{Q}$, the smaller the performance degradation caused by discretization. In addition, the value set of the context-aware weight is denoted by W. Then, the state space S={Q×W} is thus countable but infinite.
- Action space: At each slot, the vehicle can take two actions, namely U(t)∈U={0,1}, where U(t)=1 denotes the vehicle deciding to transmit updates in slot *t* and U(t)=0 denotes the vehicle deciding to wait.
- Probability transfer function: After taking action *U* at state s=(Q,ω), the next state is denoted by $s^{\prime }=\left(Q^{\prime },\omega^{\prime }\right)$. When the vehicle decides not to transmit or the transmission fails, the probability of the estimation error transferring from *Q* to $Q^{\prime }$ is written as $\mathrm{Pr}\left\{Q^{\prime }-Q=a\right\}=p_{a}$. Due to the discretization of the estimation error, the increment $a\in A=\{0,\pm \Delta_{Q},\pm 2\Delta_{Q},\cdots ,\pm A_{m}\}$, where $A_{m}=\left\lfloor \frac{A_{max}}{\Delta_{Q}}\right\rfloor \Delta_{Q}>0$. In addition, $p_{a}=F_{A}\left(a+\frac{1}{2}\Delta_{Q}\right)-F_{A}\left(a-\frac{1}{2}\Delta_{Q}\right)$, where $F_{A}\left(a\right)$ is the CDF of increment A(t). In addition, the probability of the context-aware weight transferring from ω to $\omega^{\prime }$ is written as $\mathrm{Pr}\left\{\omega \to \omega^{\prime }\right\}=p_{\omega \omega^{\prime }}$. Based on the assumption that the context-aware weight ω(t) is independent with the estimation error Q(t), then the probability of the state transferring from s=(Q,ω) to $s^{\prime }=\left(Q^{\prime },\omega^{\prime }\right)$ given action *U* is:
  (6) $$\begin{array}{cc} \; & \mathrm{Pr}\left\{s\to s^{\prime }|U\right\}=\mathrm{Pr}\left\{\left(Q,\omega \right)\to \left(Q^{\prime },\omega^{\prime }\right)|U\right\}\\ \; & =\left\{\begin{array}{cc} p_{\omega \omega^{\prime }}p_{Q^{\prime }-Q} & ,U=0,\\ p_{\omega \omega^{\prime }}\left(\left(1-p_{s}\right)p_{Q^{\prime }-Q}+p_{s}p_{Q^{\prime }-0}\right) & ,U=1. \end{array}\right. \end{array}$$
- One-step cost: The cost caused by taking action *U* in state (Q,ω) is:
  (7) $$\begin{array}{c} C\left(Q,\omega ,U\right)=\omega Q^{2}, \end{array}$$
  while the one-step updating penalty only depends on the chosen action:
  (8) $$\begin{array}{c} D(Q,\omega ,U)=U. \end{array}$$

The average cost caused under a certain policy π is the average UoI, which is defined as ${\bar{C}}^{\pi }$ and the average updating penalty under π is defined as ${\bar{D}}^{\pi }$. We aimed to find the UoI-optimal policy which minimizes the average cost under the resources constraint. Therefore, problem (5) can be formulated into the following CMDP problem:

(9) $$\begin{array}{cc} \min_{\pi } & \;{\bar{C}}^{\pi }=\lim_{T\to \infty }\frac{1}{T}E_{\pi }\left[\sum_{t=1}^{T}C\left(Q\left(t\right),\omega \left(t\right),U\left(t\right)\right)\right]\\ s.t. & \;{\bar{D}}^{\pi }=\lim_{T\to \infty }\frac{1}{T}E_{\pi }\left[\sum_{t=1}^{T}D\left(Q\left(t\right),\omega \left(t\right),U\left(t\right)\right)\right]\leq \rho . \end{array}$$

### 3.2. Threshold Structure of the Optimal Policy

We start from some basic definitions in [33] and show the properties of problem (9).

**Definition** **1.** *A stationary deterministic policy is a policy that takes the same action whenever in a given state s=(Q,ω), while a stationary randomized policy chooses to update or not in state s with a certain probability.*

**Theorem** **1.** *There exists an optimal stationary randomized policy for problem (9). The optimal policy is a probabilistic combination of two stationary deterministic policies. The two deterministic policies only differ on at most one state and each policy minimizes the unconstrained cost in (10) with a different Lagrange multiplier λ:*

(10) $$\begin{array}{c} L_{\lambda }^{\pi }=\lim_{T\to \infty }\frac{1}{T}E_{\pi }\left[\sum_{t=1}^{T}\left[C(Q(t),\omega (t),U(t))+\lambda D(Q(t),\omega (t),U(t))\right]\right]. \end{array}$$

**Proof of Theorem** **1.** The proof is shown in Appendix A. □

We denote the optimal policy that minimizes the unconstrained cost in (10) with a given λ by $\pi^{★}$ and the cost obtained under policy $\pi^{★}$ by $L_{\lambda }^{\pi^{★}}$, namely $L_{\lambda }^{\pi^{★}}=\min_{\pi }L_{\lambda }^{\pi }$. Then, there exists a differential cost function V(Q,ω) that satisfies the Bellman Equation [34]:

(11) $$\begin{array}{cc} \; & V\left(Q,\omega \right)+L_{\lambda }^{\pi^{★}}=\min\{C\left(Q,\omega ,1\right)+\lambda D\left(Q,\omega ,1\right)\\ & +\left(1-p_{s}\right)\sum_{\omega^{\prime }\in W}p_{\omega \omega^{\prime }}\sum_{a=-A_{m}}^{A_{m}}p_{a}V\left(Q+a,\omega^{\prime }\right)+p_{s}\sum_{\omega^{\prime }\in W}p_{\omega \omega^{\prime }}\sum_{a=-A_{m}}^{A_{m}}p_{a}V\left(a,\omega^{\prime }\right),\\ & C\left(Q,\omega ,0\right)+\sum_{\omega^{\prime }\in W}p_{\omega \omega^{\prime }}\sum_{a=-A_{m}}^{A_{m}}p_{a}V\left(Q+a,\omega^{\prime }\right)\}. \end{array}$$

To solve problem (5), we first prove that with a given λ, the optimal stationary deterministic policy $\pi^{★}$ has a threshold structure. We then introduce a discounted problem with a discount factor α and the discounted cost starting from state (Q,ω) under a certain policy π is:

(12) $$\begin{array}{cc} J_{\alpha ,\pi }\left(Q,\omega \right)= & \lim_{T\to \infty }E_{\pi }[\sum_{t=0}^{T}\alpha^{t}\left[C(Q(t),\omega (t),U(t))\right.\\ \; & \left.+\lambda D(Q(t),\omega (t),U(t))\right]|\left(Q\left(0\right)=Q,\omega \left(0\right)=\omega \right)]. \end{array}$$

Denote the minimum cost starting from state (Q,ω) by $V_{\alpha }\left(Q,\omega \right)=\min_{\pi }J_{\alpha ,\pi }\left(Q,\omega \right)$. Then, we have:

(13) $$\begin{array}{cc} V_{\alpha }\left(Q,\omega \right)= & \min\{C\left(Q,\omega ,1\right)+\lambda D\left(Q,\omega ,1\right)+\left(1-p_{s}\right)\alpha \sum_{\omega^{\prime }\in W}p_{\omega \omega^{\prime }}\sum_{a=-A_{m}}^{A_{m}}p_{a}V_{\alpha }\left(Q+a,\omega^{\prime }\right)\\ + & p_{s}\alpha \sum_{\omega^{\prime }\in W}p_{\omega \omega^{\prime }}\sum_{a=-A_{m}}^{A_{m}}p_{a}V_{\alpha }\left(a,\omega^{\prime }\right),C\left(Q,\omega ,0\right)\\ + & \alpha \sum_{\omega^{\prime }\in W}p_{\omega \omega^{\prime }}\sum_{a=-A_{m}}^{A_{m}}p_{a}V_{\alpha }\left(Q+a,\omega^{\prime }\right)\}. \end{array}$$

Define Δ(Q,ω) as the difference between the value functions by taking the two different actions U=0,1, meaning that:

(14) $$\begin{array}{cc} \; & \Delta \left(Q,\omega \right)=C\left(Q,\omega ,0\right)+\alpha \sum_{\omega^{\prime }\in W}p_{\omega \omega^{\prime }}\sum_{a=-A_{m}}^{A_{m}}p_{a}V_{\alpha }\left(Q+a,\omega^{\prime }\right)\\ - & C\left(Q,\omega ,1\right)-\lambda D\left(Q,\omega ,1\right)-p_{s}\alpha \sum_{\omega^{\prime }\in W}p_{\omega \omega^{\prime }}\sum_{a=-A_{m}}^{A_{m}}p_{a}V_{\alpha }\left(a,\omega^{\prime }\right)\\ - & \left(1-p_{s}\right)\alpha \sum_{\omega^{\prime }\in W}p_{\omega \omega^{\prime }}\sum_{a=-A_{m}}^{A_{m}}p_{a}V_{\alpha }\left(Q+a,\omega^{\prime }\right)\\ = & p_{s}\alpha \sum_{\omega^{\prime }\in W}p_{\omega \omega^{\prime }}\sum_{a=-A_{m}}^{A_{m}}p_{a}\left\{V_{\alpha }\left(Q+a,\omega^{\prime }\right)-V_{\alpha }\left(a,\omega^{\prime }\right)\right\}-\lambda . \end{array}$$

Define $\sum_{a=-A_{m}}^{A_{m}}p_{a}V_{\alpha }\left(Q+a,\omega \right)$ as a function $f_{\alpha }\left(Q,\omega \right)$. Then we will prove that for $\forall |Q_{1}\left|<\right|Q_{2}|$, we have $f_{\alpha }\left(Q_{1},\omega \right)<f_{\alpha }\left(Q_{2},\omega \right)$. To this end, we first prove the following Lemma 1.

**Lemma** **1.** *For a given discount factor α and a fixed context-aware weight ω, the value function for Q equals the value function for −Q, namely:*

$$V_{\alpha }\left(Q,\omega \right)=V_{\alpha }\left(-Q,\omega \right).$$

**Proof of Lemma** **1.** The Lemma is proven by induction. Define $V_{\alpha }^{\left(k\right)}\left(Q,\omega \right)$ as the value function obtained after the $k^{\mathrm{th}}$ iteration. Assume that for ∀Q, we have: $V_{\alpha }^{\left(k\right)}\left(Q,\omega \right)=V_{\alpha }^{\left(k\right)}\left(-Q,\omega \right)$. If action *U* is taken in the $k^{\mathrm{th}}$ iteration, then the expected discounted cost is defined as $J_{\alpha ,U}^{\left(k\right)}\left(Q,\omega \right)$. Therefore, $V_{\alpha }^{\left(k+1\right)}\left(Q,\omega \right)=\min_{U}J_{\alpha ,U}^{\left(k\right)}\left(Q,\omega \right)$. We have:

(15) $$\begin{array}{cc} J_{\alpha ,0}^{\left(k\right)}\left(Q,\omega \right)\; & =C\left(Q,\omega ,0\right)+\alpha \sum_{\omega^{\prime }\in W}p_{\omega \omega^{\prime }}\sum_{a=-A_{m}}^{A_{m}}p_{a}V_{\alpha }^{\left(k\right)}\left(Q+a,\omega^{\prime }\right)\\ \; & =\omega {\left(-Q\right)}^{2}+\alpha \sum_{\omega^{\prime }\in W}p_{\omega \omega^{\prime }}\sum_{a=-A_{m}}^{A_{m}}p_{a}V_{\alpha }^{\left(k\right)}\left(-Q-a,\omega^{\prime }\right)\\ \; & =C_{X}\left(-Q,\omega ,0\right)+\alpha \sum_{\omega^{\prime }\in W}p_{\omega \omega^{\prime }}\sum_{a=-A_{m}}^{A_{m}}p_{a}V_{\alpha }^{\left(k\right)}\left(-Q+a,\omega^{\prime }\right)=J_{\alpha ,0}^{\left(k\right)}\left(-Q,\omega \right). \end{array}$$

Similarly, we can further prove that $J_{\alpha ,1}^{\left(k\right)}\left(Q,\omega \right)=J_{\alpha ,1}^{\left(k\right)}\left(-Q,\omega \right)$. Notice that the value function obtained in ${\left(k+1\right)}^{\mathrm{th}}$ iteration is obtained by: $V_{\alpha }^{\left(k+1\right)}\left(Q,\omega \right)=\min_{U}J_{\alpha ,U}^{\left(k\right)}\left(Q,\omega \right),$ and for any action *U*, $J_{\alpha ,U}^{\left(k\right)}\left(Q,\omega \right)=J_{\alpha ,U}^{\left(k\right)}\left(-Q,\omega \right)$. Thus, $V_{\alpha }^{\left(k+1\right)}\left(Q,\omega \right)=V_{\alpha }^{\left(k+1\right)}\left(-Q,\omega \right)$. By letting k→∞, $V_{\alpha }^{\left(k\right)}\left(Q,\omega \right)\to V_{\alpha }\left(Q,\omega \right)$. Hence, $V_{\alpha }\left(Q,\omega \right)=V_{\alpha }\left(-Q,\omega \right)$. □

**Lemma** **2.** *For a given discount factor α and a fixed context-aware weight ω, function $f_{\alpha }\left(Q,\omega \right)$ for Q increases monotonically with the absolute value of Q, namely: for $\forall |Q_{1}\left|<\right|Q_{2}|,f_{\alpha }\left(Q_{1},\omega \right)<f_{\alpha }\left(Q_{2},\omega \right)$.*

**Proof of Lemma** **2.** Using the induction method, we first assume that for $\forall |Q_{1}\left|<\right|Q_{2}|$, we have $f_{\alpha }^{\left(k\right)}\left(Q_{1},\omega \right)<f_{\alpha }^{\left(k\right)}\left(Q_{2},\omega \right)$. Therefore:

(16) $$\begin{array}{cc} J_{\alpha ,0}^{\left(k\right)}\left(Q_{1},\omega \right)\; & =C\left(Q_{1},\omega ,0\right)+\alpha \sum_{\omega^{\prime }\in W}p_{\omega \omega^{\prime }}\sum_{a=-A_{m}}^{A_{m}}p_{a}V_{\alpha }^{\left(k\right)}\left(Q_{1}+a,\omega^{\prime }\right)\\ \; & =\omega Q_{1}^{2}+\alpha \sum_{\omega^{\prime }\in W}p_{\omega \omega^{\prime }}f_{\alpha }^{\left(k\right)}\left(Q_{1},\omega^{\prime }\right)\\ \; & <C\left(Q_{2},\omega ,0\right)+\alpha \sum_{\omega^{\prime }\in W}p_{\omega \omega^{\prime }}f_{\alpha }^{\left(k\right)}\left(Q_{2},\omega^{\prime }\right)\\ & =J_{\alpha ,0}^{\left(k\right)}\left(Q_{2},\omega \right). \end{array}$$

Similarly, we can obtain $J_{\alpha ,1}^{\left(k\right)}\left(Q_{1},\omega \right)<J_{\alpha ,1}^{\left(k\right)}\left(Q_{2},\omega \right)$. Meanwhile, $V_{\alpha }^{\left(k+1\right)}\left(Q,\omega \right)=\min_{U}J_{\alpha ,U}^{\left(k\right)}\left(Q,\omega \right)$, then we have $V_{\alpha }^{\left(k+1\right)}\left(Q_{1},\omega \right)<V_{\alpha }^{\left(k+1\right)}\left(Q_{2},\omega \right)$, for $\forall |Q_{1}\left|<\right|Q_{2}|$. Obviously, if we want to use induction to complete the proof of Lemma 2, we have to prove that: $f_{\alpha }^{\left(k+1\right)}\left(Q_{1},\omega \right)<f_{\alpha }^{\left(k+1\right)}\left(Q_{2},\omega \right)$, for $\forall |Q_{1}\left|<\right|Q_{2}|$. To simplify the proof, it is assumed that $Q_{2}>Q_{1}>0$. The discussion will be divided into the following three situations.

- When $A_{m}\leq \left|Q_{1}\right|$, then $|Q_{1}+a|<|Q_{2}+a|$, for $\forall a\in [-A_{m},A_{m}]$, we can derive that:
  (17) $$\begin{array}{cc} f_{\alpha }^{\left(k+1\right)}\left(Q_{1},\omega \right)= & \sum_{a=-A_{m}}^{A_{m}}p_{a}V_{\alpha }^{\left(k+1\right)}\left(Q_{1}+a,\omega \right)\\ < & \sum_{a=-A_{m}}^{A_{m}}p_{a}V_{\alpha }^{\left(k+1\right)}\left(Q_{2}+a,\omega \right)=f_{\alpha }^{\left(k+1\right)}\left(Q_{2},\omega \right). \end{array}$$
- When $A_{m}>\left|Q_{2}\right|$, there exists an increment $a^{\prime }\in A^{\prime }=\left\{a\right|a\in \left[-A_{m},-\frac{1}{2}\left(Q_{1}+Q_{2}\right)\right\}$, such that $|Q_{1}+a^{\prime }\left|>\right|Q_{2}+a^{\prime }|$, and $V_{\alpha }^{\left(k+1\right)}\left(Q_{1}+a^{\prime },\omega^{\prime }\right)>V_{\alpha }^{\left(k+1\right)}\left(Q_{2}+a^{\prime },\omega^{\prime }\right)$. Notice that $-Q_{1}-a^{\prime }\in \left(\frac{1}{2}\left(Q_{2}-Q_{1}\right),A_{m}-Q_{1}\right]$ and $Q_{2}+a\in \left[-A_{m}+Q_{2},A_{m}+Q_{2}\right]$, then $p_{-Q_{1}-a^{\prime }-Q_{2}}V_{\alpha }^{\left(k+1\right)}\left(-Q_{1}-a^{\prime },\omega \right)$ is a term in the summation $f_{\alpha }^{\left(k+1\right)}\left(Q_{2},\omega \right)$, namely $\sum_{a=-A_{m}}^{A_{m}}p_{a}V_{\alpha }\left(Q+a,\omega \right)$. Similarly, $p_{-Q_{2}-a^{\prime }-Q_{1}}V_{\alpha }^{\left(k+1\right)}\left(-Q_{2}-a^{\prime },\omega \right)$ is a term in the summation $f_{\alpha }^{\left(k+1\right)}\left(Q_{1},\omega \right)$. We further define $A^{\prime \prime }=\left\{a|a=-Q_{1}-Q_{2}-a^{\prime }\right\}$, since $-Q_{1}-Q_{2}-a^{\prime }\in \left(-\frac{1}{2}\left(Q_{1}+Q_{2}\right),A_{m}-Q_{1}-Q_{2}\right]$, then $A^{\prime }\cap A^{\prime \prime }=⌀$.
  Furthermore, the probability of the estimation error transferring from $Q_{1}$ to $-Q_{2}-a^{\prime }$, i.e., $p_{-Q_{2}-a^{\prime }-Q_{1}}$ equals $p_{-Q_{1}-a^{\prime }-Q_{2}}$, the probability of the estimation error transferring from $Q_{2}$ to $-Q_{1}-a^{\prime }$. Since $-a^{\prime }\in \left(\frac{1}{2}\left(Q_{1}+Q_{2}\right),A_{m}\right]$, then $|a^{\prime }\left|>\right|-Q_{1}-Q_{2}-a^{\prime }|$. According to our assumption of the increment, we can prove that for any $a^{\prime }\in A^{\prime }$, $p_{a^{\prime }}<p_{-Q_{1}-Q_{2}-a^{\prime }}$. Then, we can derive:
  (18) $$\begin{array}{cc} \; & f_{\alpha }^{\left(k+1\right)}\left(Q_{1},\omega \right)-f_{\alpha }^{\left(k+1\right)}\left(Q_{2},\omega \right)\\ = & \sum_{a\in A^{\prime }}p_{a}V_{\alpha }^{\left(k+1\right)}\left(Q_{1}+a,\omega \right)+\sum_{a\in A^{\prime \prime }}p_{a}V_{\alpha }^{\left(k+1\right)}\left(Q_{1}+a,\omega \right)\\ - & \sum_{a\in A^{\prime }}p_{a}V_{\alpha }^{\left(k+1\right)}\left(Q_{2}+a,\omega \right)-\sum_{a\in A^{\prime \prime }}p_{a}V_{\alpha }^{\left(k+1\right)}\left(Q_{2}+a,\omega \right)+M\left(Q_{1},Q_{2}\right)\\ = & \sum_{a\in A^{\prime }}p_{a}\left\{V_{\alpha }^{\left(k+1\right)}\left(Q_{1}+a,\omega \right)-V_{\alpha }^{\left(k+1\right)}\left(Q_{2}+a,\omega \right)\right\}\\ + & \sum_{a\in A^{\prime }}p_{-Q_{1}-Q_{2}-a}\left\{V_{\alpha }^{\left(k+1\right)}\left(Q_{2}+a,\omega \right)-V_{\alpha }^{\left(k+1\right)}\left(Q_{1}+a,\omega \right)\right\}+M\left(Q_{1},Q_{2}\right)\\ = & \sum_{a\in A^{\prime }}\left(p_{a}-p_{-Q_{1}-Q_{2}-a}\right)\left\{V_{\alpha }^{\left(k+1\right)}\left(Q_{1}+a,\omega \right)-V_{\alpha }^{\left(k+1\right)}\left(Q_{2}+a,\omega \right)\right\}+M\left(Q_{1},Q_{2}\right)<0, \end{array}$$
  where $M\left(Q_{1},Q_{2}\right)=\sum_{a\notin A^{\prime }\cup A^{\prime \prime }}p_{a}\left(V_{\alpha }^{\left(k+1\right)}\left(Q_{1}+a,\omega \right)-V_{\alpha }^{\left(k+1\right)}\left(Q_{2}+a,\omega \right)\right)<0$.
- When $|Q_{2}|>A_{m}>\left|Q_{1}\right|$, since $a^{\prime }\in \left[-A_{m},-\frac{1}{2}\left(Q_{1}+Q_{2}\right)\right)$, we only need to consider the case when $A_{m}>\frac{1}{2}\left(Q_{1}+Q_{2}\right)$, in this case $-Q_{1}-a^{\prime }>\frac{1}{2}\left(Q_{2}-Q_{1}\right)>Q_{2}-A_{m}$. Therefore, $p_{-Q_{1}-a^{\prime }-Q_{2}}V_{\alpha }^{\left(k+1\right)}\left(-Q_{1}-a^{\prime },\omega \right)$ is a term in the summation $f_{\alpha }^{\left(k+1\right)}\left(Q_{2},\omega \right)$. Similarly, we can also prove that $f_{\alpha }^{\left(k+1\right)}\left(Q_{1},\omega \right)<f_{\alpha }^{\left(k+1\right)}\left(Q_{2},\omega \right)$ when $|Q_{2}|>A_{m}>\left|Q_{1}\right|$.

According to Lemma 1, the conclusions above can be easily generalized to the cases without the condition $Q_{2}>Q_{1}>0$. Finally, by letting k→∞, $V_{\alpha }^{\left(k+1\right)}\left(Q,\omega \right)\to V_{\alpha }\left(Q,\omega \right)$, therefore: $f_{\alpha }^{\left(k+1\right)}\left(Q,\omega \right)\to f_{\alpha }\left(Q,\omega \right)$. Hence: $f_{\alpha }\left(Q_{1},\omega \right)<f_{\alpha }\left(Q_{2},\omega \right)$. □

**Remark** **1.** *Lemma 2 holds when $f_{A}\left(a\right)$, i.e., the PDF of increment A(t) satisfies the following conditions:*

- *$f_{A}\left(a\right)=f_{A}\left(-a\right)$, μ=0;*
- *$f_{A}\left(a_{2}\right)\leq f_{A}\left(a_{1}\right),\forall a_{2}\geq a_{1}\geq 0$.*

Then, with Lemmas 1 and 2, we can prove the threshold structure of the optimal stationary deterministic policy which minimizes $L_{\lambda }^{\pi }$ in (10).

**Theorem** **2.** *For a given λ, the optimal stationary deterministic policy which minimizes $L_{\lambda }^{\pi }$ in (10) has a threshold structure when the context-aware weight is a first-order irreducible positive recurrent Markov process.*

**Proof of Theorem** **2.** Let $s_{\alpha }^{*}\left(Q,\omega \right)$ denote the optimal action which minimizes the discounted cost $V_{\alpha }\left(Q,\omega \right)$ at state (Q,ω). If the optimal action $s_{\alpha }^{*}\left(Q,\omega \right)=1$, then the vehicle will transmit its status update to the center at state (Q,ω) and Δ(Q,ω)≥0. Thus, we have:

(19) $$\begin{array}{c} \Delta \left(Q,\omega \right)=p_{s}\alpha \sum_{\omega^{\prime }\in W}p_{\omega \omega^{\prime }}\sum_{a=-A_{m}}^{A_{m}}p_{a}\left\{V_{\alpha }\left(Q+a,\omega^{\prime }\right)-V_{\alpha }\left(a,\omega^{\prime }\right)\right\}-\lambda \geq 0. \end{array}$$

According to Lemma 2, for any $|Q^{\prime }\left|>|Q\right|$, $\Delta (Q^{\prime },\omega )$ can be lower bounded by

(20) $$\begin{array}{cc} \Delta (Q^{\prime },\omega ) & =p_{s}\alpha \sum_{\omega^{\prime }\in W}p_{\omega \omega^{\prime }}\sum_{a=-A_{m}}^{A_{m}}p_{a}\left\{V_{\alpha }\left(Q^{\prime }+a,\omega^{\prime }\right)-V_{\alpha }\left(a,\omega^{\prime }\right)\right\}-\lambda \\ \; & \geq p_{s}\alpha \sum_{\omega^{\prime }\in W}p_{\omega \omega^{\prime }}\sum_{a=-A_{m}}^{A_{m}}p_{a}\left\{V_{\alpha }\left(Q+a,\omega^{\prime }\right)-V_{\alpha }\left(a,\omega^{\prime }\right)\right\}-\lambda \geq 0. \end{array}$$

If Δ(Q,ω)>0, then for any states with $|Q^{\prime }\left|>|Q\right|$, the optimal policy is to transmit the status to the center. If Δ(Q,ω)<0, then for any states with $|Q^{\prime }\left|<|Q\right|$, the optimal action is not to transmit. In addition, the optimal policy will not be choosing to wait in all the slots. Therefore, for each context-aware weight ω, there must be a threshold $\tau_{\omega }\geq 0$. For any state (Q,ω) with $|Q|>\tau_{\omega }$, the optimal choice is to transmit the status update. We can then conclude that for a given weight ω, the optimal policy with a discount factor α has a threshold structure.

Let $\left\{\alpha_{1},\alpha_{2},\cdots ,\alpha_{k}\right\}$ denote a sequence of discount factors and $\alpha_{k}$ converges to 1 when k→∞. Then, the optimal deterministic policy for α=1 will also converge to the optimal policy with a discount factor which is less than 1 [35]. Similar derivation is also applied in [12]. Therefore, we can prove the threshold structure of the optimal stationary deterministic policy which minimizes $L_{\lambda }^{\pi }$. □

Similarly, when the context-aware weight is i.i.d. over time, we can obtain the following theorem:

**Theorem** **3.** *For a given λ, the optimal stationary deterministic policy which minimizes $L_{\lambda }^{\pi }$ in (10) has a threshold structure when the context-aware weight is i.i.d. over time. The thresholds are the same for each state of the context-aware weight.*

**Proof of Theorem** **3.** If the context-aware weight is i.i.d. over time, then we have:

(21) $$\begin{array}{c} \Delta \left(Q,\omega \right)=p_{s}\alpha \sum_{\omega^{\prime }\in W}p_{\omega^{\prime }}\sum_{a=-A_{m}}^{A_{m}}p_{a}\left\{V_{\alpha }\left(Q+a,\omega^{\prime }\right)-V_{\alpha }\left(a,\omega^{\prime }\right)\right\}-\lambda =\Delta \left(Q\right), \end{array}$$

where $p_{\omega }$ is the probability of the value of the context-aware weight being in state ω. Therefore, in this case, the state will be reduced to one dimension and the thresholds will be the same for all the states of the context-aware weight. □

According to Theorems 2 and 3, we proved the threshold structure of the two stationary deterministic policies that compose the UoI-optimal policy. Since the UoI-optimal policy for problem (9) is a probabilistic combination of two deterministic policies with threshold structures, we can finally draw the conclusion that the UoI-optimal policy also has a threshold structure.

### 3.3. Numerical Solution of Optimal Strategy

Based on Theorem 2, we only need to consider the policy that chooses to update with a probability of 1 in state (Q,ω), for $\forall |Q|\geq Q_{\max}=\max_{\omega }\tau_{\omega }$. Let $\mu_{Q,\omega }$ denote the probability that the state of the vehicle is (Q,ω). $y_{Q,\omega }$ denotes the probability that the state is (Q,ω) and the vehicle chooses to transmit an update. Therefore, we have:

**Theorem** **4.** *When the context-aware weight is a first-order irreducible positive recurrent Markov process, the UoI-optimal policy can be derived by solving the following LP problem:*

(22a) $$\begin{array}{cc} \{\mu_{Q,\omega }^{*},y_{Q,\omega }^{*}\}= & \arg\min_{\left\{\mu_{Q,\omega },y_{Q,\omega }\right\}}\sum_{\omega \in W}\sum_{Q=-Q_{\max}}^{Q_{\max}}\omega Q^{2}\mu_{Q,\omega }, \end{array}$$

(22b) $$\begin{array}{cc} \;s.t.\; & \sum_{\omega \in W}\sum_{Q=-Q_{\max}}^{Q_{\max}}\mu_{Q,\omega }=1, \end{array}$$

(22c) $$\begin{array}{cc} \; & \sum_{\omega \in W}\sum_{Q=-Q_{\max}}^{Q_{\max}}y_{Q,\omega }\leq \rho , \end{array}$$

(22d) $$\begin{array}{cc} \; & y_{Q,\omega }\leq \mu_{Q,\omega },\forall Q,\omega , \end{array}$$

(22e) $$\begin{array}{cc} \; & 0\leq y_{Q,\omega }\leq 1,0\leq \mu_{Q,\omega }\leq 1,\forall Q,\omega ,\\ \; & \mu_{Q,\omega }=\sum_{\omega^{\prime }\in W}\sum_{Q^{\prime }=-Q_{\max}}^{Q_{\max}}y_{Q^{\prime },\omega^{\prime }}p_{s}p_{Q}p_{\omega \omega^{\prime }} \end{array}$$

(22f) $$\begin{array}{c} +\sum_{\omega^{\prime }\in W}\sum_{Q^{\prime }=-Q_{\max}}^{Q_{\max}}\left(\mu_{Q^{\prime },\omega^{\prime }}-y_{Q^{\prime },\omega^{\prime }}p_{s}\right)p_{Q^{\prime }-Q}p_{\omega \omega^{\prime }}. \end{array}$$

**Proof of Theorem** **4.** We first derive the average UoI ${\bar{C}}^{\pi }$ as a function of $\mu_{Q,\omega }$ and $y_{Q,\omega }$. The vehicle is in state (Q,ω) and produces a cost of $C\left(Q,\omega ,u\right)=\omega Q^{2}$ with a probability of $\mu_{Q,\omega }$. Therefore, the average UoI is:

(23) $$\begin{array}{c} \sum_{\omega \in W}\sum_{Q=-Q_{\max}}^{Q_{\max}}\omega Q^{2}\mu_{Q,\omega }. \end{array}$$

As for the constraints, (22b) means that the sum of the probabilities of all the states should be 1. To explain (22c), we note that $y_{Q,\omega }$ is the probability of the vehicle being in state (Q,ω) and choosing to transmit the update, then the expectation of a one-step updating penalty for state (Q,ω) in (8) is $\mu_{Q,\omega }$. Therefore, the constraint on average update frequency ${\bar{D}}^{\pi }$ can be illustrated by

(24) $$\begin{array}{c} \sum_{\omega \in W}\sum_{Q=-Q_{\max}}^{Q_{\max}}\mu_{Q,\omega }\leq \rho . \end{array}$$

Then, we introduce $\xi_{Q,\omega }\in \left[0,1\right]$ to represent that the probability of the vehicle choosing to transmit updates in state (Q,ω) and (22d) can be obtained by the fact that $y_{Q,\omega }=\mu_{Q,\omega }\xi_{Q,\omega }$, while (22e) is derived by the nature of probability.

The right-hand side of (22f) can be viewed as two terms. The first term is the sum of transition probability from all the states to state (Q,ω) when the vehicle chooses to update and the transmission of status is successful. The second term is the sum of transition probability from all the states to state (Q,ω) when the transmission is failed or the vehicle chooses to wait. Therefore, we can prove that the optimal solution of problem (5) equals the solution of the LP problem. □

When ω(t) is i.i.d. over time, we can also obtain the UoI-optimal policy through the LP problem proposed in Theorem 4 and only need to use $p_{\omega^{\prime }}$ as a replacement of $p_{\omega \omega^{\prime }}$.

## 4. Scheduling in Unknown Contexts

To make decisions, the UoI-optimal updating policy obtained in Section 3 still needs the distributions of the context-aware weight ω(t), the increment A(t) and the successful transmission probability, which may not be available in advance or may change over time in most practical systems. To solve this problem, we will assume that the distribution of the context-aware weight is not pre-determined and the vehicle has to learn it. In this section, we use the reinforcement learning (RL) algorithm to learn the dynamic of the context and the characteristic of the wireless channel.

To solve this problem, we turn to the model-based RL framework proposed in [36]. We only consider the cases when the UoI-optimal policy has a threshold structure. This assumption makes the optimal policy based on the truncated state space equal the optimal policy of the original problem.

We use the 3-tuple $\left(s,s^{\prime },U\right)$ to formulate the proposed RL-based updating policy. The states in the current slot and next slot are denoted by *s* and $s^{\prime }$, respectively. *U* denotes the action chosen in the current slot. The settings of the discretized state space and the action space are the same as the settings proposed in Section 3.1. The smaller the step size used in the discretization is, the closer our results are to those in continuous state space. In addition, the selection of the step size only affects the accuracy of the update threshold. Therefore, the performance loss caused by discretization can be reduced by choosing a smaller step size.

We display details about the proposed RL-based updating policy in Algorithm 1. At the beginning of episode *k*, we randomly decide whether to explore or exploit. l∈[0,1] represents the trade-off between exploration and exploitation during the following episode. A larger *l* means a higher frequency of exploration and vice versa. If the algorithm chooses to explore during this episode, a random policy $\pi_{rand}\left(s\right)$ will be used, i.e., we randomly choose to update or not in each state to find more valuable actions. If the algorithm chooses to exploit, then we have to obtain the probability transfer functions ${\tilde{p}}_{k}\left(s^{\prime }|s,U\right)$ for each state transmission pair. In Algorithm 1, N(s,U) and $N(s,U,s^{\prime })$ represent the number of occurrences of state–action pair s,U and state transition from *s* to $s^{\prime }$ given action *U*, respectively. Based on the assumption that the optimal policy has a threshold structure, the policy π(k) which can minimize the average UoI with the estimated probability transfer functions, can be directly solved through the LP problem proposed in Theorem 4. Then, the vehicle will use policy $\pi_{k}$ to derive state–action pairs and the state transitions in the following $\left\lceil L_{k}\right\rceil$ slots. Here, L>0 is defined to control the number of state transitions observed in each episode. At the end of each episode, the model will be updated according to the state–action pairs and the state transitions observed during the episode. Finally, after *K* episodes, the algorithm will output the RL-based updating policy $\pi^{★}\left(s\right)$, which is derived based on ${\tilde{p}}_{K}\left(s^{\prime }|s,U\right)$.
**Algorithm 1** RL-based Updating Policy**Input:**  l∈[0,1],L>0,K>01:**for** episodes k=1,2,⋯,K **do**2: Set $L_{k}=L\sqrt{k},\epsilon_{k}=l/\sqrt{k}$, uniformly draw α∈[0,1].3: **if** $\alpha <\epsilon_{k}$ **then**4:  Set $\pi_{k}\left(s\right)=\pi_{rand}\left(s\right),$5: **else**6:  **for** each state $s,s^{\prime }\in S$ and U∈U **do**7:   **if** N(s,U)>0 **then**8:    Let ${\tilde{p}}_{k}\left(s^{\prime }|s,U\right)=N\left(s,U,s^{\prime }\right)/N\left(s,u\right),$9:   **else**10:    ${\tilde{p}}_{k}\left(s^{\prime }|s,U\right)=1/\left|S\right|.$11:   **end if**12:  **end for**13:  obtain policy $\pi_{k}\left(s\right)$ by solving the estimated CMDP14: **end if**15: Randomly choose an initial state s(1).16: **for** slots $t=1,2,\cdots ,\lceil L_{k}\rceil -1$ **do**17:  Choose action U(t) as $\pi_{k}\left(s\left(t\right)\right)$.18:  Observe the next state s(t+1).19:  N(s(t),U(t),s(t+1))=N(s(t),U(t),s(t+1))+1.20:  N(s(t),U(t))=N(s(t),U(t))+1.21:  s(t)←s(t+1).22: **end for**23:**end for**24:obtain policy $\pi^{★}\left(s\right)$ by solving the estimated CMDP based on ${\tilde{p}}_{k}\left(s^{\prime }|s,U\right)$, $s,s^{\prime }\in S,U\in U$.**Output:** output the RL-based updating policy $\pi^{★}\left(s\right)$

## 5. Simulation Results and Discussion

### 5.1. Simulation Setup

**Figure 2:**
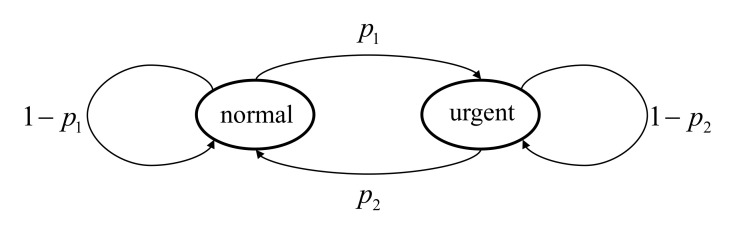
The state transition diagram of ω(t).

To facilitate the simulation, we consider the case where the context-aware weight of the vehicle only has two different states: the ’normal’ state and ’urgent’ state. The ’normal’ state means that the vehicle is in ordinary situations and the significance of accuracy of status information is relatively low. We set ω(t) as 1 in ’normal’ state while ω(t) is set as a constant much larger than 1, $\omega_{e}$, in ’urgent’ state to show that the vehicle is in emergencies. Two different distributions of the context-aware weight are taken into consideration to conform to the assumptions about ω(t) used in Section 3.1:

1. The context-aware weight ω(t) has the first-order Markov property. The state transition diagram of ω(t) is shown in Figure 2 and ω(t) is irreducible and positive recurrent. $p_{1}$ is the probability of the context-aware weight transferring from the normal state to the urgent state, while $p_{2}$ is the probability of the weight transferring from the urgent state to the normal state;
2. The context-aware weight ω(t) is i.i.d. over time. The probability of the weight being in the urgent state and the normal state are denoted by $p_{h}$ and $p_{l}$, respectively.

As for the increment A(t), $A_{max}$ is set to a large enough positive number to simplify the simulations.

### 5.2. Numerical Results

**Figure 3:**
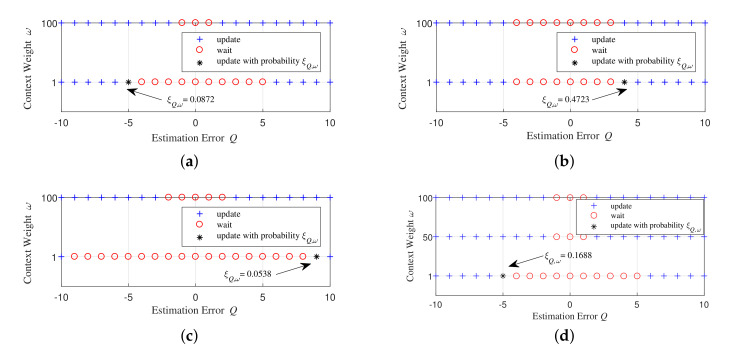
Threshold structure of the UoI-optimal updating policy when: (**a**) the context-aware weight is a first-order Markov process, $\rho =0.05,p_{1}=0.001,p_{2}=0.01,p_{s}=0.9,\sigma^{2}=1,\omega_{e}=100$; (**b**) the context-aware weight is i.i.d. over time, $\rho =0.05,p_{l}=0.999,p_{h}=0.001,p_{s}=0.9,\sigma^{2}=1,\omega_{e}=100$; (**c**) the context-aware weight is a first order Markov process, $\rho =0.05,p_{1}=0.001,p_{2}=0.01,p_{s}=0.9,\omega_{e}=100$, increment in the estimation error during one slot, i.e., A(t)∼Unif(−3,3), for ∀t; and (**d**) the context-aware weight is a three-state first-order Markov process, which takes value from $\omega_{1}=1,\omega_{2}=50,\omega_{3}=100$ and evolves according to the state transition matrix $P_{3}$, $\rho =0.05,p_{s}=0.9,\sigma^{2}=1$.

Figure 3 shows the structure of the UoI-optimal updating policy. For the discretization of the estimation error, the step size used is 1. It can be seen that under the two different distributions of the context-aware weight mentioned above, the optimal updating policies all have threshold structures. Especially when the context-aware weight is i.i.d. over time, Figure 3b shows that thresholds for all the states of the context-aware weight are the same, which matches well with theoretical analysis. From Figure 3c, we can find that the UoI-optimal policy also has threshold structure when increment A(t) obeys a uniform distribution Unif(−3,3), which verifies Remark 1. We then simulate the UoI-optimal policy under the contexts with more states to show the policy is generic. We consider a three-state context-aware weight which takes value from $\omega_{1}=1,\omega_{2}=50,\omega_{3}=100$. The state transition matrix $P_{3}$ of the three-state context-aware weight is:

(25) $$P_{3}=\left[\begin{array}{ccc} 0.997 & 0.002 & 0.001\\ 0.02 & 0.97 & 0.01\\ 0.2 & 0.1 & 0.7 \end{array}\right],$$

where the *j*-th element on the *i*-th row indicates the probability that the context transfers from state $\omega_{i}$ to state $\omega_{j}$. The numerical results (Figure 3d) show that when the context-aware weight has more states, the UoI-optimal policy still has a threshold structure, which verifies our theoretical results.

**Figure 4:**
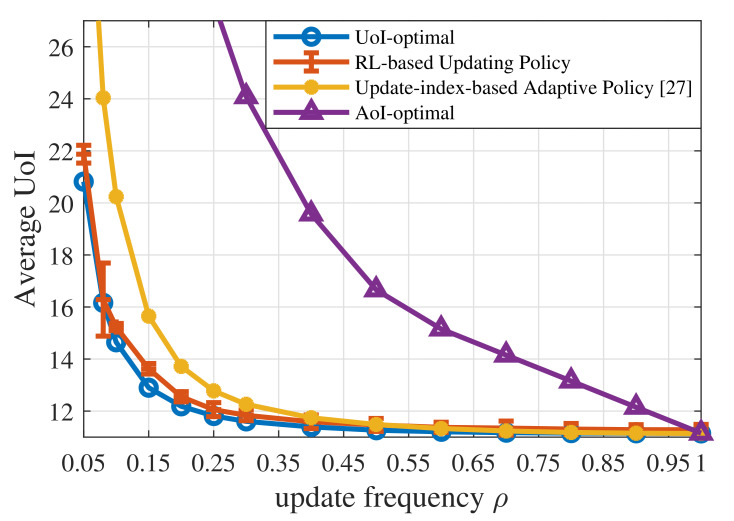
Average UoI of the UoI-optimal updating policy, the RL-based updating policy, the update-index-based adaptive scheme [27], and the AoI-optimal updating policy when $p_{1}=0.001,p_{2}=0.01,p_{s}=0.9,\sigma^{2}=1,\omega_{e}=100$.

Then, we will focus on the results obtained when the context-aware weight is a first-order irreducible positive recurrent Markov process, as shown in Figure 2. Figure 4 shows the average UoI of the UoI-optimal policy, the AoI-optimal policy derived by CMDP, the RL-based updating policy, and the update-index-based adaptive scheme [27]. In the RL-based updating policy, L=8000, l=1 and K=50. All the numerical results of the RL-based policy are averaged over 100 runs.

First of all, the UoI-optimal policy can only be obtained based on advanced information about the system dynamics. However, the RL-based policy achieves near-optimal without knowing the system dynamics, which indicates that Algorithm 1 learns relatively accurate probability transfer functions from the observed state–action pairs and state transitions during the training.

**Figure 5:**
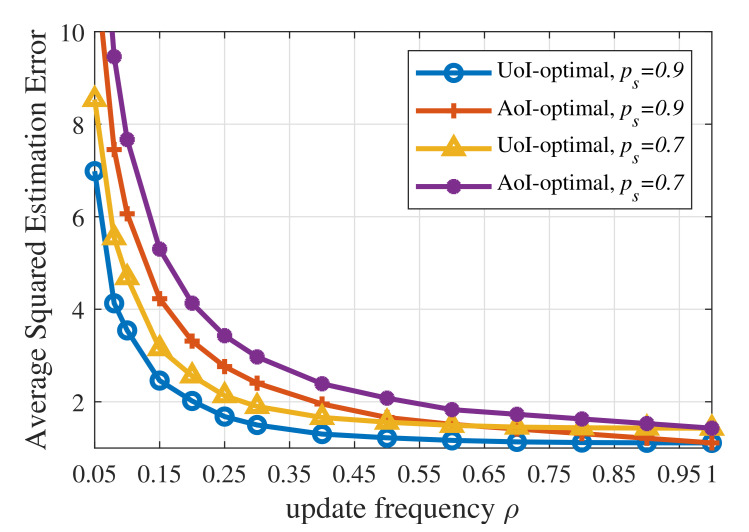
Average squared estimation error of the UoI-optimal updating policy and the AoI-optimal updating policy when $p_{1}=0.001,p_{2}=0.01,\sigma^{2}=1,\omega_{e}=100$.

Secondly, according to Figure 4, the AoI-optimal policy yields a much higher UoI than the three UoI-based policies, namely the UoI-optimal policy, the RL-based updating policy, and the update-index-based adaptive scheme. On the one hand, AoI is one special case of UoI. When the context-aware weight ω(t)=1, the increment A(t)=1, and the cost function δ(Q(t))=Q(t), then UoI equals AoI. Therefore, the AoI-optimal policy ignores the fact that different contexts have different requirements for information freshness. In the proposed UoI-based updating policies, different contexts have different policies and update thresholds, while the AoI-optimal updating policies for different contexts are the same. On the other hand, Figure 5 reveals that the AoI-optimal policy leads to a much higher estimation error, which results in worse performance in terms of UoI. The AoI-optimal policy is an oblivious policy, which is independent of the monitored process. Since AoI increases linearly with time, the AoI-optimal policy can only minimize the linear performance degradation in terms of time. However, the UoI-based policies (the cost function $\delta \left(Q\left(t\right)\right)={\left(Q\left(t\right)\right)}^{2}$) considered in this paper are process-dependent, which are called non-oblivious policies, and can benefit from both age and process realization [37]. These policies can directly minimize the nonlinear impact exerted by information staleness and the gap between the actual status and the estimated status.

Thirdly, our updating policies outperform the update-index-based adaptive scheme [27] in terms of UoI. Under the adaptive scheme, the vehicle will derive an update index as a function of the current estimation error and the context-aware weight for the next slot. If the index is larger than the adaptive update threshold, then the vehicle is supposed to transmit its status information to the center. If the vehicle transmits an update in slot *t*, then the adaptive threshold will increase in the next slot; otherwise, the adaptive threshold will decrease. The adaptive scheme will cause an overuse of the resource in `urgent’ states and lead to the fact that the vehicles cannot receive resources in `normal’ states. However, the UoI-optimal policy and the trained RL-updating policy are fixed schemes, which can avoid the extremely unbalanced resource allocation between the two contexts and achieve better performance.

**Figure 6:**
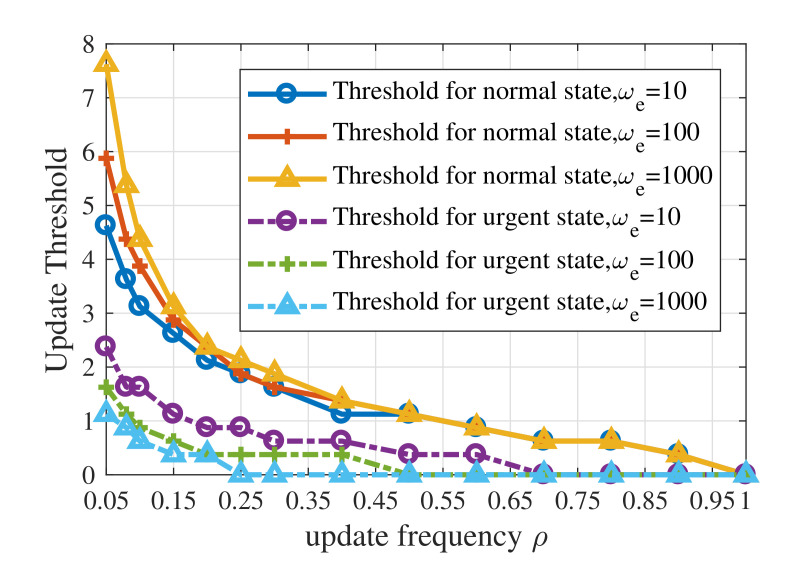
Update thresholds of the UoI-optimal updating policy with different values of $\omega_{e}$ when $p_{1}=0.001,p_{2}=0.01,p_{s}=0.9,\sigma^{2}=1$.

Figure 6 shows the influence of the maximum average update frequency ρ and the context weight for emergency, $\omega_{e}$, on update threshold of UoI-optimal policy. In order to obtain more accurate results, the step size used here is 0.25. The solid curves show update thresholds for the normal state while the dashed curves show update thresholds for the urgent state. When the constraint on update resources is strict, the update thresholds fall faster. Furthermore, a larger $\omega_{e}$ results in a lower update threshold for the urgent state and a higher threshold for the normal state. This phenomenon indicates that the value of $\omega_{e}$ means the tolerance of estimation error in the emergency. When ρ<0.1, the influence of $\omega_{e}$ on the update threshold for the normal state is larger than the urgent state. For the cases where the maximum average update frequency is relatively large, $\omega_{e}$ has little effect on update thresholds for both normal state and urgent state.

**Figure 7:**
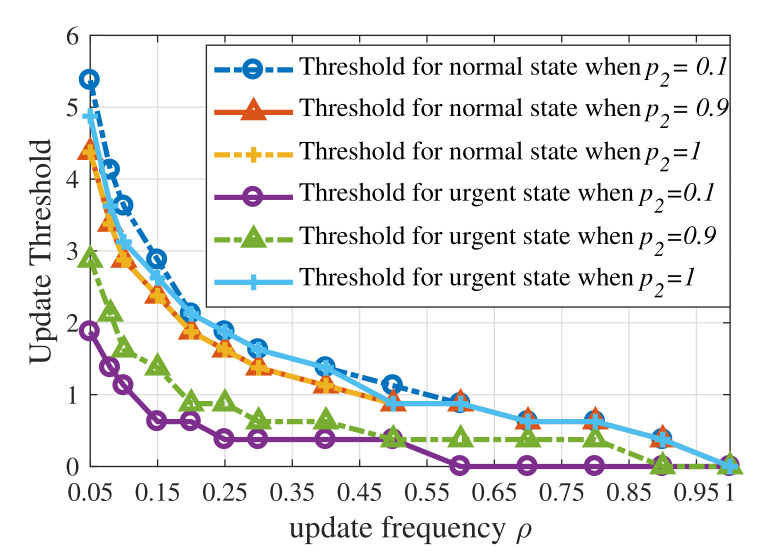
Update thresholds of the UoI-optimal updating policy with different values of $p_{2}$ when $p_{1}=0.01,p_{s}=0.9,\sigma^{2}=1,\omega_{e}=100$.

Figure 7 shows that the update thresholds also depend on the dynamic of context-aware weight when the weight has first-order Markov property. When $p_{2}$ is approaching $1-p_{1}$, the gap between update thresholds for the urgent state and the normal state becomes smaller for the context-aware weight which tends to be i.i.d. over time. When $p_{2}=1$, the update threshold for the urgent state exceeds the threshold for the normal state. Therefore, the update threshold for the urgent state is not necessarily lower than the update threshold for the normal state.

**Figure 8:**
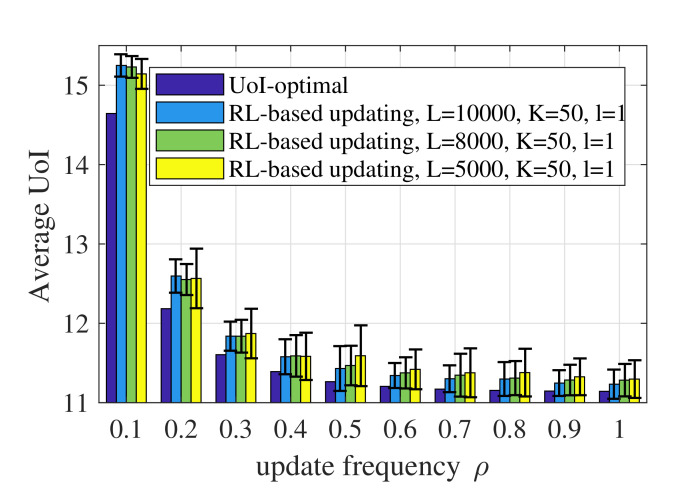
Average UoI of the RL-based updating policy with different values of *L* when $p_{1}=0.001,p_{2}=0.01,\sigma^{2}=1,\omega_{e}=100$.

Figure 8 shows the performance of the RL-based updating policy with different values of *L*. According to Algorithm 1, the number of state transitions observed in episode *k* is $\left\lceil L\sqrt{k}\right\rceil$. Therefore, *L* denotes the number of state transitions observed during the whole learning process. Generally speaking, a larger *L* reduces the randomness of the performance and achieves a better UoI. The performance of the RL-based updating policy depends on the accuracy of the model obtained through training, namely whether the estimated probability transfer function of the system is accurate. A larger *L* means that the algorithm can collect more data or state transitions and obtain a more accurate model.

**Figure 9:**
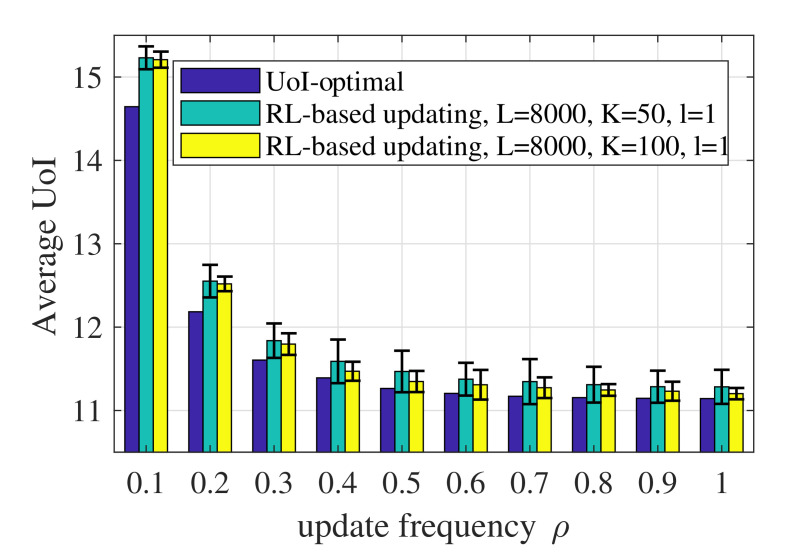
Average UoI of the RL-based updating policy with different values of *K* when $p_{1}=0.001,p_{2}=0.01,\sigma^{2}=1,\omega_{e}=100$.

Figure 9 shows the influence of the number of episodes, i.e., *K*, on the performance of the RL-based updating policy. A larger *K* leads to a lower average UoI and smaller randomness over 100 runs. On the one hand, the more episodes and the more data the algorithm observes, the more accurate the model obtained will be and the better the performance of the updating policy will be. On the other hand, the value of *K* is the number of iterations for the policy obtained through the estimated CMDP. The policy $\pi_{k}\left(s\right)$ used in episode *k* is derived based on the state–action pairs and the state transitions observed in the previous k−1 episodes. Therefore, more frequent iterations of the updating policy can obtain more valuable state–action pairs and better performance.

## 6. Conclusions

In this work, we studied how to minimize the performance degradation caused by outdated information in terms of UoI, which is a new metric jointly considering context and information freshness. We proved that the UoI-optimal updating policy for the considered single-user remote monitoring system has a single threshold structure. Then, the policy was obtained through linear programming by assuming that the state transition probability of the system is known in advance. In unknown contexts, we further used a reinforcement learning algorithm to learn the dynamics of the system. Simulations verified the threshold structure of the UoI-optimal policies and showed that the update thresholds decrease as the maximum average update frequency increases. In addition, a larger context-aware weight in emergencies resulted in a lower update threshold for urgent states. However, since the state transition probability also influenced the update thresholds, the update threshold for emergencies was not necessarily higher than the update threshold for normal states, especially when the probability of transferring from urgent states to normal states tended towards 1. Furthermore, the numerical results showed that the proposed RL-based updating policy achieved a near-optimal performance without advanced knowledge of the system model.

In fact, determining the context-aware weight in practical systems, where the models of the context are often very complicated and difficult to obtain in advance, remains open. As for future work, we plan to use deep RL algorithms to learn the models of the context variation. We believe that UoI can provide a new performance metric for information timeliness measurement in the future V2X scenario. In addition, we believe the proposed UoI metric and the context-aware scheduling policy can shed some light on low-latency and ultra-reliable wireless communication in the future 5G/6G systems.

## Data Availability

Not applicable.

## Abbreviations

The following abbreviations are used in this manuscript:

AoI	Age of Information
AoII	Age of Incorrect Information
AoS	Age of Synchronization
CDF	Cumulative Distribution Function
CMDP	Constrained Markov Decision Process
CoUD	Cost of Update Delay
i.i.d.	Independent and Identically Distributed
IoT	Internet of Things
LP	Linear Programming
PDF	Probability Density Function
RL	Reinforcement Learning
UoI	Urgency of Information
URLLC	Ultra-Reliable Low-Latency Communication
VR	Virtual Reality
V2X	Vehicle to Everything

## Appendix A. Proof of Theorem 1

Given a state s=(Q,ω)∈S and a nonempty subset of the state space, G⊂S, let R(s,G) denote the class of policies θ such that the probability $P^{\theta }\left(s\left(t\right)\in G\;\mathrm{for}\;\mathrm{some}\;t\geq 1|s\left(0\right)=s\right)=1$ and the expected time $m_{s,G}\left(\theta \right)$ of the first passage from *s* to G under policy θ is finite. Then, let $R^{★}\left(s,G\right)$ denote the class of policies θ such that the expected average UoI $c_{s,G}\left(\theta \right)$ and the expected transmission cost $d_{s,G}\left(\theta \right)$ of the first passage from *s* to G are finite and θ∈R(s,G). To prove Theorem 1, we then introduce Assumptions A1–A5 in [33]:

**Assumption** **A1.**
*For all b>0, the set G(b)≜{s|thereexistsanactionUsuchthatC(s,U)+D(s,U)≤b} is finite.*

**Assumption** **A2.**
*There exists a stationary deterministic policy π that induces a Markov chain with the following properties: the state space consists of a single (nonempty) positive recurrent class $R^{\pi }$ and a set $T^{\pi }$ of transient states such that $\pi \in R^{★}\left(s,R^{\pi }\right)$, for any $s\in T^{\pi }$, and both the average UoI ${\bar{C}}^{\pi }$ and the average transmission cost ${\bar{D}}^{\pi }$ on $R^{\pi }$ are finite.*

**Assumption** **A3.**
*Given any two states $s,s^{\prime }\in S$ and $s\neq s^{\prime }$, there exists a policy π (a function of s and $s^{\prime }$) such that $\pi \in R^{★}\left(s,\left\{s^{\prime }\right\}\right)$.*

**Assumption** **A4.**
*If a stationary deterministic policy has at least one positive recurrent state, then it has a single positive recurrent class, and this class contains the state (Q,ω) with Q=0.*

**Assumption** **A5.**
*There exists a policy π such that the average UoI ${\bar{C}}^{\pi }<\infty$ and average transmission cost ${\bar{D}}^{\pi }<\rho$.*

Furthermore, the problem (9) has the following property:

**Lemma** **A1.**
*Assumptions A1–A5 hold for problem (9).*

**Proof of Lemma** **A1.** First of all, we focus on the cases where the context-aware weight is assumed as a first-order irreducible positive recurrent Markov process:

- Assumption A1: In this problem, C(s,U) is the UoI at state *s*, namely $C\left(Q,\omega ,U\right)=\omega Q^{2}$. D(s,U) is 1 if the vehicle chooses to transmit its status and D(s,U) is 0 otherwise, namely D(Q,ω,U)=U. Therefore, Assumption A1 holds, for any b>0, the number of states (Q,ω) with $\omega Q^{2}\leq b$ is finite.
- Assumption A2: Due to the current high-level wireless communication technology, we reasonably assumed that the successful transmission probability $p_{s}$ is relatively close to 1. Based on the assumptions mentioned above, the Markov chain of context-aware weight obviously satisfies Assumption A2. Define the probability of the context-aware weight transferring from ω to $\omega^{\prime }$ in *k* steps for the first time as $P_{\omega ,\omega^{\prime },k}$. Then, we consider the policy π(Q,ω)=1 for all (Q,ω)∈S, namely this policy chooses to transmit in all the states.
  Since the evolution of the context-aware weight is independent with the evolution of the estimation error and the updating policy. Therefore, we first focused on the estimation error, which can be formulated as a one-dimensional irreducible Markov chain with state space $Q=\{0,\pm \Delta_{Q},\pm 2\Delta_{Q},\cdots ,\pm n\Delta_{Q},\cdots \}$. We denote the set of states which can transfer to state *Q* in a single step by $Z_{Q}$. The probability of the estimation error transferring from state *Q* to state $Q^{\prime }$ at the *k*-th step without an arrival to state Q=0 is defined as $P_{Q,Q^{\prime },k}^{\prime }$. Obviously, $\sum_{Q^{\prime }\in Q}P_{Q,Q^{\prime },k}^{\prime }<{\left(1-p_{s}\right)}^{k}$. Then, the probability of the first passage from state Q(Q≠0) to 0 taking k+1 steps is $\sum_{Q^{\prime }\notin Z_{0}}P_{Q,Q^{\prime },k}^{\prime }p_{s}+\sum_{Q^{\prime }\in Z_{0}}P_{Q,Q^{\prime },k}^{\prime }\left(p_{s}+\left(1-p_{s}\right)p_{0-Q^{\prime }}\right)<{\left(1-p_{s}\right)}^{k}$, where $p_{0-Q^{\prime }}$ is the probability that the increment in estimation error is $-Q^{\prime }$. Therefore, the expected time of the first passage from Q(Q≠0) to 0 is finite.
  For state Q=0, the estimation error will stay in this state in the next step with a probability of $p_{s}+p_{0-0}$ and will first return to state Q=0 in the second transition with a probability smaller than $\left(1-p_{s}-p_{0-0}\right)$. Then, starting from state Q=0, the estimation error will first return to state Q=0 in the k+1-th (k>2) step will be smaller than $\left(1-p_{s}-p_{0-0}\right){\left(1-p_{s}\right)}^{k-1}$. Therefore, we can prove that state Q=0 is a positive recurrent state, and $R_{Q}^{\pi }=\left\{Q=0\right\}$ is a positive recurrent class of the induced Markov chain of the estimation error. Furthermore, for any states in $T_{Q}^{\pi }=Q\setminus R_{Q}^{\pi }$, the expected time of the first passage from the state in $T_{Q}^{\pi }$ to state Q=0 under π is finite and the probability of the states in $T_{Q}^{\pi }$ not getting to state Q=0 in *k* steps is smaller than ${\left(1-p_{s}\right)}^{k}$.
  Define the probability of state *Q* transferring to state $Q^{\prime }$ in *k* steps for the first time as $P_{Q,Q^{\prime },k}$. Then, the probability of state (Q,ω) transferring to state $\left(Q^{\prime },\omega^{\prime }\right)$ in *k* steps for the first time is $P_{Q,Q^{\prime },k}P_{\omega ,\omega^{\prime },k}$. Since $\sum_{k=1}^{\infty }P_{Q,Q^{\prime },k}k<\infty$ and $\sum_{k=1}^{\infty }P_{\omega ,\omega^{\prime },k}k<\infty$, then $\sum_{k=1}^{\infty }P_{Q,Q^{\prime },k}P_{\omega ,\omega^{\prime },k}k<\infty$. Therefore, the set of states $R^{\pi }=\left\{\left(Q,\omega \right)|Q\in R_{Q}^{\pi },\omega \in W\right\}$ is a positive recurrent class. Similarly, we can prove that $T^{\pi }=S\setminus R^{\pi }$ satisfies Assumption A2. Finally, ${\bar{D}}^{\pi }=1<\infty$, ${\bar{C}}^{\pi }=E\left[\omega \right]\frac{1}{p_{s}}\sigma^{2}<\infty$.
- Assumption A3: Define $P_{Q,\min}=\min_{Q^{\prime }}p_{Q-Q^{\prime }}$, $P_{Q,max}=\max_{Q^{\prime }}p_{Q-Q^{\prime }}$. Consider the policy $\pi^{\prime }\left(Q,\omega \right)=0$ for all states (Q,ω)∈S, notably that this policy chooses not to transmit in any states. Similarly, we first focus on the Markov chain of estimation error. Starting from state *Q*, the probability of transferring to state $Q^{\prime }$ in k+1-th (k≥2) steps for the first time is smaller than $\left(1-p_{Q^{\prime }-Q}\right)P_{Q^{\prime },max}{\left(1-P_{Q^{\prime },min}\right)}^{k-1}$. Then, the expected time of the first passage from state *Q* to state $Q^{\prime }$ under policy $\pi^{\prime }$ is finite. Similarly, since the Markov chain of context-aware weight is irreducible positive recurrent and independent with the updating policy, we can therefore prove that the expected time of the first passage from state (Q,ω) to state $\left(Q^{\prime },\omega^{\prime }\right)$ under policy $\pi^{\prime }$ is finite.
- Assumption A4: For the Markov chain of the estimation error, any state will return to state Q=0 if a successful transmission occurs. For the policy without transmission, namely $\pi^{\prime }\left(Q,\omega \right)=0$, state Q=0 still exists in only one positive recurrent class. For each positive recurrent class containing state Q=0, we can prove that there is only one positive recurrent class. Since the Markov chain of the context-aware weight is irreducible positive recurrent, we can similarly prove Assumption A4.
- Assumption A5: The policy $\pi_{\rho }$ that updates the status with a probability of ρ−δ satisfies Assumption A5. Here, δ is a small positive number. Under this policy, ${\bar{D}}^{\pi }=\rho -\delta <\rho$ and ${\bar{C}}^{\pi }=E\left[\omega \right]\frac{1}{p_{s}\left(\rho -\delta \right)}\sigma^{2}<\infty$.

Similarly, we can prove that Assumptions A1–A5 also holds for problem (9) when the context-aware weight is i.i.d. over time. □

Since Assumptions A1–A5 hold for problem (9), then according to Theorem 2.5 in [33], there exists an optimal stationary randomized policy for problem (9). Meanwhile, the optimal policy is a probabilistic combination of two stationary deterministic policies which only differ on at most one state.

Furthermore, according to Lemma 3.9 in [33], the two stationary deterministic policies each optimize the unconstrained cost in (10) with a different λ.
